# Identification of sequence variants associated with severe microtia-astresia by targeted sequencing

**DOI:** 10.1186/s12920-019-0475-x

**Published:** 2019-01-28

**Authors:** Pu Wang, Yibei Wang, Xinmiao Fan, Yaping Liu, Yue Fan, Tao Liu, Chongjian Chen, Shuyang Zhang, Xiaowei Chen

**Affiliations:** 1 Department of Otolaryngology, Peking Union Medical College Hospital, Beijing, China; 2 Department of Medical Genetics, School of Basic Medicine, Chinese Academy of Medical Sciences and Peking Union Medical College, Beijing, China; 3 College of Informatics, Huazhong Agricultural University, Wuhan, Hubei Province China; 4 Department of Cardiology, Peking Union Medical College Hospital, Peking Union Medical College and Chinese Academy of Medical Sciences, Beijing, China

**Keywords:** Severe microtia-atresia, Next-generation sequencing, Association analysis

## Abstract

**Background:**

Microtia-atresia is characterized by abnormalities of the auricle (microtia) and aplasia or hypoplasia of the external auditory canal, often associated with middle ear abnormalities. To date, no causal genetic mutations or genes have been identified in microtia-atresia patients.

**Methods:**

We designed a panel of 131 genes associated with external/middle or inner ear deformity. Targeted genomic capturing combined with next-generation sequencing (NGS) was utilized to screen for mutations in 40 severe microtia-atresia patients. Mutations detected by NGS were filtered and validated. And then mutations were divided into three categories—rare or novel variants, low-frequency variants and common variants—based on their frequency in the public database. The rare or novel mutations were prioritized by pathogenicity analysis. For the low-frequency variants and common variants, we used association studies to explore risk factors of severe microtia-atresia.

**Results:**

Sixty-five rare heterozygous mutations of 42 genes were identified in 27 (67.5%) severe microtia-atresia patients. Association studies to determine genes that were potentially pathogenic found that *PLEC*, *USH2A*, *FREM2*, *DCHS1*, *GLI3*, *POMT1* and *GBA* genes were significantly associated with severe microtia-atresia. Of these, *DCHS1* was strongly suggested to cause severe microtia-atresia as it was identified by both low-frequency and common variants association studies. A rare mutation (c.481C > T, p.R161C) in *DCHS1* identified in one individual may be deleterious and may cause severe microtia-atresia.

**Conclusion:**

We identified several genes that were significantly associated with severe microtia-atresia. The findings provide new insights into genetic background of external ear deformities.

**Electronic supplementary material:**

The online version of this article (10.1186/s12920-019-0475-x) contains supplementary material, which is available to authorized users.

## Background

Severe microtia-atresia, one of the most frequent congenital craniofacial deformities, is characterized by abnormalities of the auricle (microtia) and aplasia or hypoplasia of the external auditory canal, often associated with middle ear abnormalities [1, 2]. The prevalence of microtia-atresia has been reported to vary from 0.83 to 4.34 per 10,000 [3]. It can occur unilaterally (79–93%) or bilaterally [2–4], with approximately 80% of unilateral microtia-atresia cases occurring on the right side [5, 6]. Microtia-atresia occurs more frequently in males, with an estimated 20–40% increased risk compared with females [2, 5, 7]. Severe microtia-atresia affects not only the appearance of children, but also psychological status and hearing [8]. The identification of genetic risk factors or pathogenic genes for severe microtia-atresia is helpful to find its etiology and prevention strategies.

During embryonic development, complex tissue interactions are needed to form the external, middle, and inner ear. The external ear consists of the auricle, the external auditory canal and the outer layer of the tympanic membrane, which derives from the space between the first and second branchial arch in the developing embryo [9]. The external ear begins its development during the fifth week, and the hillocks are first identifiable during the sixth week of embryogenesis. There are six hillocks that surround the first pharyngeal cleft—the space between the first and second arches; each hillock contributes to a specific component of the pinna [10]. The auricular hillocks grow, fuse, and undergo morphogenesis to produce the external ear, a progress that lasts several months and takes place largely during fetal stages. The auricles migrate from their initially low position on the embryonic neck to their normal anatomical position [11]. Any risk factor that affects external ear development will lead to microtia during embryonic development.

Pathogenic genes of most types of syndromic microtia have been determined, but the etiology of nonsyndromic microtia-atresia is very complicated. Strong evidence supports the involvement of genetic and environmental factors, as well as their interaction, in the disease. Epidemiological studies of microtia have indicated various environmental risk factors, such as viral infections, poisoning and anemia during pregnancy; diabetes; and high maternal age or maternal medication usage [2, 4]. Familial clustering of microtia can generate an optimal research model for genetic exploration, especially for monogenic disease. Estimates of the familial incidence of microtia range from 3 to 34%, and both autosomal-dominant and autosomal-recessive patterns of inheritance have been reported [12–14]. Animal model and human genetic studies have revealed several genomic regions or genes associated with syndromic and non-syndromic microtia, namely trisomy chromosomal 13 and 18, 6p24, 4p duplication, *HOXA1*/*A2*, *SIX1*/*SIX4*/*SIX5*, *EYA1*, *TBX1*, *IRF6*, *CHUK*, *FGF3*, *PRX1*/*PRX2*, and *GSC* [15, 16]. However, genes responsible for nonsyndromic microtia have not been identified and validated in human. Recent attempts to identify such genes include the sequencing of *PRKRA*, *PACT*, *GSC*, *HOXA2* and *SIX2* in microtia-atresia patients, which may be pathogenic genes underlying microtia-atresia, but no mutations have been identified [17–20].

To increase our knowledge about genetics of severe microtia-atresia and to decipher its genetics basis, we designed a panel containing all the published genes known to be associatedwith external/middle or inner ear development and screened for mutations in 40 severe microtia-atresia cases. The external ear deformities of all individuals were classified as grade III according to the classification by Hunter [21]. The identified genetic variants were divided into three categories—rare, low-frequency and common—and each was studied comprehensively to explore their associations with microtia-atresia.

## Methods

### Samples

Forty patients (28 males, 12 females; mean age, 8.4 years; range, 6–26 years) who presented with severe microtia-atresia at Peking Union Medical College Hospital (PUMCH) were included. All external ear deformities were classified as grade III (according to the classification by Hunter [21]) external ear deformity. Patients with syndromic forms of microtia, such as Treacher Collins syndrome, Miller syndrome, Charge syndrome and Branchio-Oto-Renal (BOR) syndrome, were excluded from the study. All patients were asked for a detailed family history, and none had a family history of microtia.

All participants or their legal guardians gave informed written consent for their blood to be taken and stored for future scientific analysis. Ethical approval for this study was obtained from the institutional review board of PUMCH (JS-796).

### Gene selection and targeted-capture design

Genes were selected based on their involvement in human diseases associated with external ear deformity and their known mutation in animal models exhibiting external ear deformities. We searched through MedGen, OMIM and ORPHA in NCBI (https://www.ncbi.nlm.nih.gov/). The following words were used for retrieval of genes associated with microtia: “microtia”, “auricle congenital malformation”, “auricle dysplasia”, “anotia”, “constricted ear”, “cup ear”, “lop ear”, and “cryptotia”. We also searched through PubMed to find out the candidate genes for microtia which were reported recently. Taking into account that some microtia patients have complicating inner ear malformations [22–25], we proposed that the development of external/middle and inner ear may be regulated by certain genes in common. Accordingly, we also selected several genes associated with inner ear deformities. Ultimately, the panel contains 131 genes associated with ear development, including 104 genes associated with external/middle ear deformities and 27 genes associated with inner ear deformities (Additional file 1). By using the SureDesign portal (https://erray.chem.agilent.com/suredesign, Agilent, USA), we designed complementary RNA capture probes against all coding exons and 25 bp of flanking intronic sequence in order to cover splice junctions of these genes.

### Library construction, target capture, and exome sequencing

Genomic DNA was extracted from peripheral blood and randomly fragmented by sonication to an average size of ~ 250 bp. A pair of Illumina sequencing adaptors was then ligated to both ends of the resulting fragments after end repair and A-tailing. DNA from different individuals was tagged by amplifying adaptor-ligated DNA products using index-tagged primers. The amplified products were purified using QIAquick PCR Purification kits (QIAGEN) and then hybridized to the custom-designed capture array targeting 1,039,379 coding bases, as per the NimbleGen’s Sequence Capture protocol for enrichment. Each target-enriched library was loaded onto a HiSeq 2500 platform, and paired-end sequencing was performed with read lengths of 125 bp, providing ~386x mean coverage depth across all samples. Raw image files were processed using base-calling software (Illumina 1.7) with default parameters.

### Read mapping, variants detection, filtering, and validation

Raw, quality-filtered sequencing reads generated by the Illumina pipeline were subjected to data pre-processing. Sequencing adaptor and low-quality sequences were discarded prior to read mapping. The high quality pair-ended reads of each sample were first aligned to the NCBI human reference genome (Hg19) using the Burrows-Wheeler Aligner with default parameters. We then performed local realignment around indels and base quality score recalibration using Genome Analysis Toolkit (GATK), removing duplicate reads using Picard tools. Single nucleotide polymorphisms (SNPs) and indels were called by the GATK package based on the improved BAM (.bam) files, as per the recommendations of the software. For subsequent analysis, we used all high-quality variants that had passed GATK quality control metrics and exhibited a coverage depth ≥ 10x. SNPs and indels were functionally annotated by ANNOVAR and categorized into missense, nonsense and splice-site mutations, and other genomic features.

To identify the most likely pathogenic mutations, we filtered out: 1) synonymous and non-coding variants (with the exception of splicing site mutations that might create an ectopic splicing site); 2) variants with an allele frequency of 0.005 or higher in the 1000 Genomes (1KG) Project, and 0.01 or higher in Exome Sequencing Project (ESP6500) and the Genome Aggregation Database (gnomAD); and 3) variants that were present in 1483 in-house controls, identified using whole-exome sequencing.

After filtration, the rare or novel mutations were verified by polymerase chain reaction (PCR) amplification and Sanger sequencing in corresponding patients. Additionally, all mutations were cross-referenced to the Human Gene Mutation Database (HGMD) to determine whether some mutations had been reported to be pathogenic.

### Association analysis

To identify other independent SNPs (common variants) that might increase microtia-atresia risk, a conditional analysis was performed on the detected variants using Plink. Because of the limited power of rare variants in an association study, we only retained SNPs with minor allele frequencies ≥0.01 in the 1KG Project. To obtain high-quality data for association testing, we pruned the discovery-stage data set in the control cohort using the following criteria: sample call rate, 99%; SNP call rate, 95%; and threshold for Hardy-Weinberg equilibrium, 0.001 (Fisher’s exact test). We extracted genotype data for Utah Residents (CEPH) with Northern and Western European Ancestry (CEU), Han Chinese in Beijing (CHB) and Southern Han Chinese (CHS) populations from the 1KG Project. A principal component analysis (PCA) was performed on these samples together with our genotyped samples using smartPCA software. Finally, the selected variants in all 40 severe microtia-atresia cases and 208 normal controls from the 1KG Project were genotyped. Tests were performed in R v3.1.1. The Bonferroni correction for multiple comparisons was applied, and the threshold for genome-wide significance was set at a *P*-value < 4.95 × 10^− 5^ (0.05/1010 variants).

To test whether low-frequency mutations (MAF < 0.01 in the 1KG Project) might also be severe microtia-atresia risk factors, a gene-based test comparing the burden of low-frequency variants in cases and controls was performed using SKAT-O implemented in the SKAT package. Only nonsynonymous variants were included in the analysis. The control individuals included in this analysis were the same as those used for common variants association tests. A Bonferroni correction was used to account for multiple testing (*P <* 7.81 × 10^− 4^).

### Molecularanalysis of variants

All rare variants identified in candidate genes were analyzed functionally. The possible impact of each mutation on the function of its respective protein was analyzed using Polyphen-2 and SIFT [26]. Structural variations in mutant candidate genes were analyzed using the HOPE server (http://www.cmbi.ru.nl/hope/) [27]. The online software program PSIPRED (v3.3) was used to predict secondary structural variations associated with potentially pathogenic mutations of candidate genes [28]. Finally, SWISS-MODEL was used to predict the tertiary structures of the proteins encoded by the *DCHS1* and *USH2A* genes, and to search three-dimensional (3D) structures deposited in the Research Collaboratory for Structural Bioinformatics Protein Data Bank (RCSB PDB) (https://swissmodel.expasy.org/). Wild-type and mutant proteins were analyzed using PyMOL software.

## Results

### Variant identification

A total of 1,039,379 bases were captured and sequenced in our study. After alignment to the reference human genome (NCBI37/hg19), 99.28% of the clean reads could be mapped to the targeted regions. The average depth for the targeted regions was 386-fold, and 91.84% of the targeted regions were covered by 20 or more reads, demonstrating the high quality of the sequencing.

### Rare or novel variants

Using the rigorous filtering pipeline, we identified 65 rare heterozygous mutations of 42 genes in 27 (67.5%) severe microtia-atresia patients (Additional file 2). Of these 65 mutations, 58 were missense mutations, 4 were non-frameshift deletions or insertions, and 3 were nonsense mutations. Of the 58 missense mutations, 23 were predicted to be deleterious to the corresponding protein by either SIFT or Polyphen-2, and 12 were predicted to be deleterious by both analysis tools. Because of limitations in the ability of in silico analysis to assess the pathogenicity of any mutation, we did not exclude mutations predicted to be benign or neutral by either SIFT or Polyphen-2. None of these mutations had been previously reported in microtia-atresia patients, and more than half (53.8%) of the variants were absent from population databases. Twenty-seven subjects (67.5%) carried at least one of these variants, and 12 out of 27 subjects (30%) carried more than one variant. Mutations in *FAT4, FKTN, FRAS1, GLI3, KMT2D, LMBRD1, PLEC, POMT1, POMT2, TP63,* and *USH2A* genes were relatively more frequent (≥2 mutations per gene) (Table 1).

**Table 1:** Mutations identified in the same gene in two or more cases

Sample ID	*FAT4*	*FKTN*	*FRAS1*	*GLI3*	*KMT2D*	*LMBRD1*	*PLEC*	*POMT1*	*POMT2*	*TP63*	*USH2A*
1454					p.P3109H	p.R479W	p.A1758V				
1467							p.G3855S				p.N336S
1549						p.G5A			p.I572V		
1575											p.N1797D
1605					p.P2193L						
1608	p.M1328 L										
1611	p.A1124V		p.Q919P						p.P730S		
1742											p.P3708L
1783										p.N568S	
XH1531					p.L3920insQQQL						
XH1535								p.L476F			
XH1546								p.L476F			p.T2807I
XH1559					p.V1561G						
XH1563				p.A1036V							
XH1566		p.I62F									p.Q3624R
XH1572										p.A502V	
XH1579			p.C3971R								p.I4386T, p.N3653S, p.V369 M
XH1582							p.S39G	p.W597X			
XH1587						p.C513R					
XH1594				p.A286V							p.R4115H
XH1745		p.H186Y									
XH1784		p.Q428X									

By searching through the HGMD database, we found 7 out of 65 rare mutations had been reported to be pathogenic in HGMD (Additional file 2). Of these, two were heterozygous mutations reported to cause related diseases: p.G310S in *TBX1* leads to DiGeorge syndrome; and p.S95dup in *TWIST1* has questionable pathogenicity, which may contribute to craniosynostosis [29–31]. Both of these diseases present phenotypes that include external ear deformity. Phenotypically, all of our patients had only external ear deformities without other deformities, differing from the phenotypes of DiGeorge syndrome and craniosynostosis. The phenotypes associated with these two mutations, however, may be affected by differences in penetrance. The remaining five mutations were homozygous or compound heterozygous mutations that can cause a related disease: *GBA* (c.1292A > G, p.N431S), which may cause Parkinson’s disease; *USH2A* (c.13157 T > C, p.I4386T and c.12344G > A, p.R4115H), which can cause Usher syndrome or retinitis pigmentosa; *SLC26A4* (c.757A > G, p.I253V), which can lead to Pendred syndrome and autosomal recessive deafness with enlarged vestibular aqueduct; and *FKTN* (c.556C > T, p.H186Y), which may be the cause of Fukuyama-type congenital muscular dystrophy (FCMD). The haploinsufficiency of these genes caused by these variants may also contribute to microtia-atresia, but this requires further study.

### Association analysis identified several genes associated with microtia-atresia

We conducted an association analysis of 40 severe microtia-atresia cases and 208 healthy controls from China. The cases and controls clustered together, and were well separated from European samples (Additional file 3: Figure S1). All Chinese samples were clustered into two subgroups, consistent with the notion of two different populations of Chinese (Northern and Southern).

**Fig. 1:**
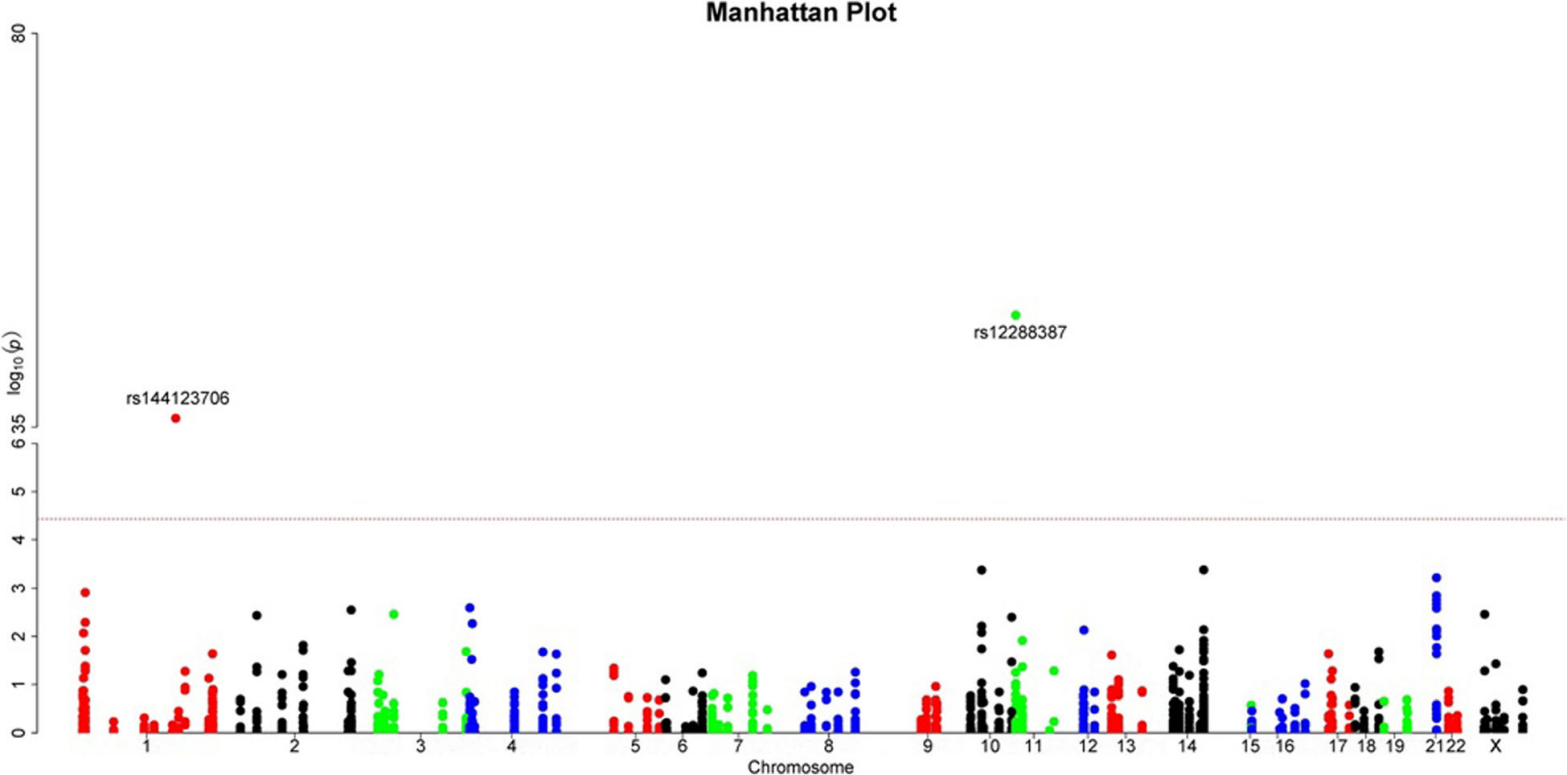
Manhattan plots of *P-*values calculated from the association of common variants. Data on 1010 single nucleotide polymorphisms (SNPs) that passed quality control criteria were collected from 40 patients with severe microtia-atresia and 208 controls. Genome-wide significance values (solid red line, *P* = 4.95 × 10^− 5^) are indicated

For common variants, we used Plink to search for single variants that might increase microtia-atresia risk. Using a Bonferroni-corrected significance level of 4.95 × 10^− 5^, two variants were identified that were significantly more common in our microtia-atresia patients: rs12288387 in *DCHS1* (dachsous cadherin-related 1) and rs144123706 in *GBA* (glucosylceramidase beta) (Fig. 1, Table 2, Additional file 4). Both variants were located in the untranslated region and were not present in other databases.

**Table 2:** Individual variant-association test results (significance level *P* = 4.95 × 10^−5^)

SNP ID	Hg19 Location	Gene	Variant	rs number	*P*-value
Chr11	6,676,884	DCHS1	NM_003737:c.-14,040 T > C	rs12288387	1.85 × 10^−48^
Chr1	155,204,694	GBA	NM_001171811:c.*92G > A	rs144123706	1.04 × 10^−36^

Notes: *indicates the mutation affects the stop codon. It makes the stop codon change to an amino acid encoded codon

For low-frequency variants, we used the SKAT-O method to explore whether the burden of these variants in any of the tested genes was higher in sporadic subjects compared with controls. After correction for multiple testing (*P* = 7.81 × 10^− 4^), *PLEC* (plectin), *USH2A* (usherin), *FREM2* (Fras1-related extracellular matrix protein 2), *DCHS1*, *GLI3* (GLI family zinc finger 3) and *POMT1* (protein O-mannosyltransferase 1) were identified as significantly associated with severe microtia-atresia (Table 3, Additional file 5).

**Table 3:** Results of gene-based, low-frequency variant association tests (significance level *P* = 7.81 × 10^−4^)

Gene	Markers	*P*-value
*PLEC*	12	1.40 × 10^−10^
*USH2A*	16	1.11 × 10^−08^
*FREM2*	6	4.38 × 10^−05^
*DCHS1*	6	0.000288
*GLI3*	5	0.000297
*POMT1*	2	0.000297

### Molecularanalysis of rare or novel variants identified in the candidate genes

**Fig. 2:**
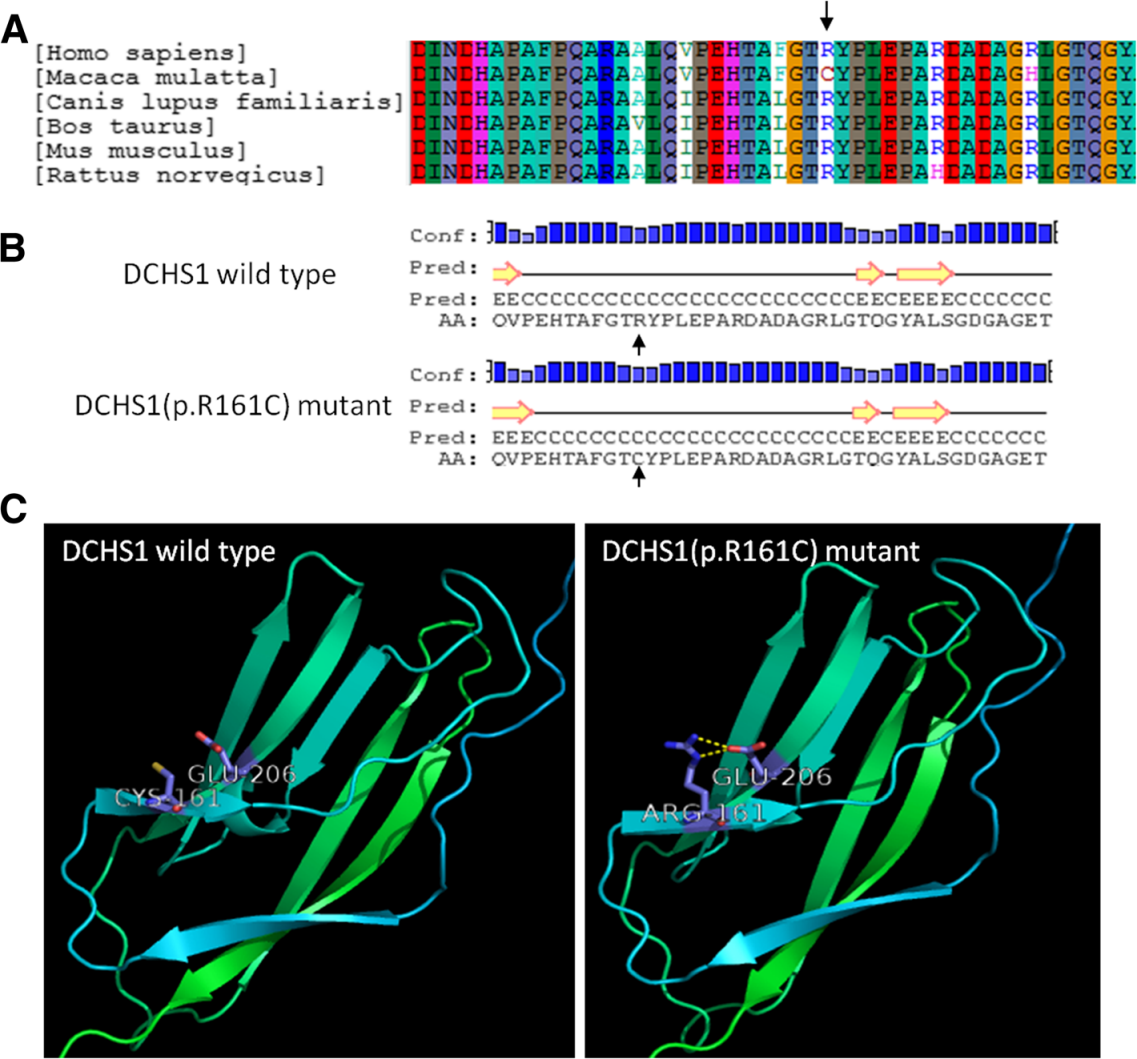
Conservation and functional analysis of the p.R161C mutation in *DCHS1*. **a** Conservation of the Arg161 residue of protocadherin-16. **b** Predicted secondary structures of the wild-type and mutant protein sequences flanking the mutations. The diagrams show the protein sequences with their secondary structures and their confidence values at the aligned positions. The secondary structure is annotated as follows: pink cylinder (alpha-helix); yellow arrow (beta-sheet); black line (coil); Conf, confidence; Pred, predictrf; H in Pred line (Helix); C in Pred line (coil); E in Pred line (sheet); AA, amino acid;, mutant amino acid. **c** Functional impact of the p.R161C mutation on the partial tertiary structure of protocadherin-16 protein predicted by molecular modeling (PDB template: 5szn.1.A; Identity 39.3%). The wild-type residue forms a salt bridge with the glutamic acid residue at position 206, with this binding damaged by substitution of the mutant residue

Further exploration of the rare mutations identified in these candidate genes suggested that these mutations be potentially etiologic. Nineteen rare mutations of seven candidate genes were identified in fourteen patients (Table 4). Both low-frequency and common-variant association studies showed that the *DCHS1* gene was significantly associated with severe microtia-atresia, suggesting a causative role for this gene. A rare mutation (c.481C > T, p.R161C) in *DCHS1* was predicted to be “damaging” by Polyphen-2 but “tolerated” by SIFT. The amino acid affected by the mutation was highly conserved among different species (Fig. 2a). HOPE server structural analysis of the protein protocadherin-16 suggested that the wild-type residue was positively charged, whereas the mutant residue was neutral. Moreover, the wild-type residue was found to be more hydrophobic than the mutant residue. This amino acid residue is located within a domain annotated in UniProt as cadherin 2. The mutation introduces an amino acid with different properties, which can disturb the cadherin 2 domain and abolish its function. Cadherin domains form homo-dimers, which are important for cell-cell interactions. A mutation in such a domain might affect these interactions. The mutation identified in this study was predicted to modify the local secondary structure of the protein (Fig. 2b). The crystal structure of the protein showed that the wild-type residue forms a salt bridge with a glutamic acid residue at position 206, and that the mutation abolished this interaction (Fig. 2c). These data indicated that the mutation is deleterious and may cause severe microtia-atresia.

**Table 4:** Rare or novel mutations identified in candidate genes by association studies

Sample ID	Gene Symbol	Consequence	GenBank accession No.	Exon	Mutation	PopFreqMax in 1000GM, ESP6500 or gnomAD	SIFT_score	SIFT_Pred	Polyphen2_HDIV_score	Polyphen2_HDIV_pred	Phylop in vertebrate	HGMD
XH1790	*DCHS1*	nonsynonymous	NM_003737	exon2	c. 481C > T, p.R161C	0.00003335	0.069	T	1	D	−0.001	NR
XH1785	*FREM2*	nonsynonymous	NM_207361	exon22	c. 8689C > T, p.R2897C	.	0	D	1	D	0.871	NR
1552	*GBA	nonsynonymous	NM_001005741	Exon10	c. 1292A > G, p.N431S	0.00001657	0.169	T	0.962	D	0.991	DM?
1454	*PLEC*	nonsynonymous	NM_201378	exon31	c.5273C > T, p.A1758V	0.0001578	0.535	T	1	D	0.897	NR
1467	*PLEC*	nonsynonymous	NM_201378	exon32	c.11563G > A, p.G3855S	0.00002367	0.652	T	0	B	−2.741	NR
	*USH2A*	nonsynonymous	NM_007123	exon6	c.1007A > G, p.N336S	.	0.048	D	0.218	B	0.991	NR
XH1582	*PLEC*	nonsynonymous	NM_000445	exon2	c. 115A > G, p.S39G	0.001536	0.381	T	0.002	B	0.991	NR
XH1535	*POMT1*	nonsynonymous	NM_001136114	exon16	c. 1428G > C, p.L476F	0.0001156	0.556	T	0.001	B	−0.043	NR
XH1546	*POMT1*	nonsynonymous	NM_001136114	exon16	c. 1428G > C, p.L476F	0.0001156	0.556	T	0.001	B	−0.043	NR
	*USH2A*	nonsynonymous	NM_206933	exon42	c. 8420C > T, p.T2807I	.	0.065	T	0.998	D	0.917	NR
XH1563	*GLI3*	nonframeshift	NM_000168	exon15	c. 3107C > T, p.A1036V	0.00007006	0.016	D	0	B	0.897	NR
XH1594	*GLI3*	nonsynonymous	NM_000168	exon7	c. 857C > T, p.A286V	.	0.18	T	0.024	B	0.917	NR
	*USH2A	nonsynonymous	NM_206933	exon63	c.12344G > A, p.R4115H	0.0005825	0.072	T	0.462	P	0.871	DM
1575	*USH2A*	nonsynonymous	NM_206933	exon27	c. 5389A > G, p.N1797D	.	1	T	0	B	−0.068	NR
1742	*USH2A*	nonsynonymous	NM_206933	exon57	c.11123C > T, p.P3708L	.	0.223	T	1	D	0.917	NR
XH1566	*USH2A*	nonsynonymous	NM_206933	exon55	c.10871A > G, p.Q3624R	.	0.271	T	0.012	B	0.079	NR
XH1579	*USH2A	nonsynonymous	NM_206933	exon63	c.13157 T > C, p.I4386T	.	0.006	D	0.978	D	1.062	DM
	*USH2A*	nonsynonymous	NM_206933	exon56	c.10958A > G, p.N3653S	.	0.409	T	0.489	P	0.991	NR
	*USH2A*	nonsynonymous	NM_007123	exon6	c. 1105G > A, p.V369 M	.	0.015	D	0.992	D	0.871	NR

Notes: *indicates the mutation has been reported in HGMD database

Abbreviations: SIFT: *D* Deleterious, *T* Tolerated; PolyPhen 2: D = Probably damaging, *B* Benign, *P* Possibly damaging; *NR* not reported, *DM*: “disease causing” mutation

**Fig. 3:**
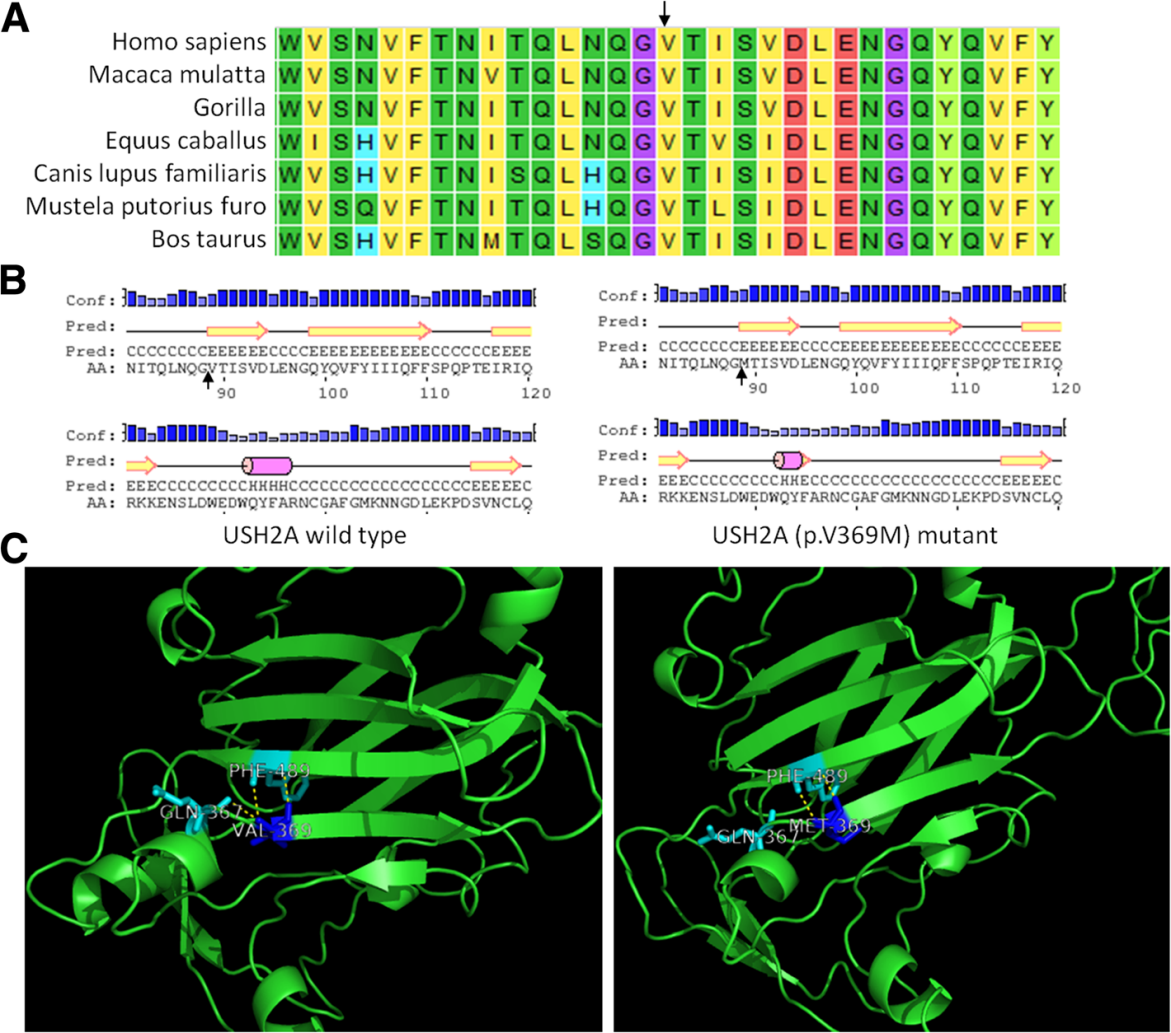
Conservation and functional analysis of the p.V369 M mutation in *USH2A*. **a** Conservation of the Val369 residue of usherin protein. **b** Predicted secondary structures of the wild-type and mutant protein sequences flanking the mutations. The diagrams show the protein sequences with their secondary structures and their confidence values at the aligned positions. The secondary structure is annotated as follows: pink cylinder (alpha-helix); yellow arrow (beta-sheet); black line (coil); Conf, confidence; Pred, predict; H in Pred line (Helix); C in Pred line (coil); E in Pred line (sheet); AA, amino acid;, mutant amino acid. **c** Functional impact of the p.V369 M mutation on the partial tertiary structure of usherin protein by molecular modeling (PDB template: 4aqs.1.A; Identity 34.9%). The wild-type residue forms two salt bridges, with glutamic acid at position 367 and with phenylalanine at position 489, both of which were damaged by the mutant residues

Patients in this study were positive for nine of the 19 rare variants found in *USH2A*. Two of these mutations (c.1105G > A, p.V369 M and c.T13157C, p.I4386T) were predicted to be “damaging” by both Polyphen-2 and SIFT, suggesting they were pathogenic of severe microtia-atresia. The amino acid affected by the first mutation, p.V369 M, is highly conserved, whereas the second, p.I4386T, is relatively conserved among different species (Fig. 3a, Additional file 6: Figure S2A). The first mutation is located within a domain annotated in UniProt as laminin N-terminal. Structural analysis of the protein usherin using the HOPE server suggested that the wild-type residue is buried within the core of the protein, whereas the mutant residue is larger and likely would not fit within the protein core, disturbing this domain and abolishing its function. The mutation was also predicted to affect the local secondary structure of the protein (Fig. 3b). The crystal structure of the protein showed that the wild-type residue forms two salt bridges, one with glutamic acid at position 367 and the other with phenylalanine at position 489, with the mutation abolishing this interaction (Fig. 3c). The second mutation, p.I4386T, is located within a domain known as fibronectin type-III 29. The wild-type residue is more hydrophobic than the mutant residue, with the Hope server showing that this mutation disturbs this domain and abolishes its function. The online PSIPRED software program also predicted that the local secondary structure of the protein was modified by this mutation (Additional file 6: Figure S2B).

PSIPRED also predicted the secondary structure of the proteins encoded by the remaining 16 mutations, as well as their respective wild-type proteins. Three mutations (p.R2897C in *FREM2*, p.A286V in GLI3, p.G3896S in *PLEC*) were predicted to modify the local secondary structure of these proteins (Additional file 7: Figure S3), although only one, p.R2897C in *FREM2*, was predicted to be “damaging” by both Polyphen-2 and SIFT. This mutation has not been reported in any database. Structural analysis of the protein encoded by *FREM2* using the HOPE server suggested that the mutation introduces a more hydrophobic residue at this position. This can result in a loss of hydrogen bonds and/or disturb correct protein folding, suggesting that this mutation may be a cause of severe microtia-atresia. In contrast, Polyphen-2 and/or SIFT predicted that the other mutations were benign.

## Discussion

Due to the complicated causes of nonsyndromic microtia-atresia, the genetic and/or environmental factors may involve in the pathogenesis of microtia. Microtia also has genetic heterogeneity and inadequate penetrance. For instance, in the reported studies of microtia family, the patients in the same family have different clinical phenotypes [16, 32–34]. In addition, we also observed in a six-generation affected 22 subjects family with non-syndromic microtia (data not yet published), the inheritance pattern is autosomal dominant. We found that some cases carried the pathogenic mutation, but the phenotype was normal. Therefore, we didn’t detect the rare or novel mutations in the parents of the related patients due to the genetic heterogeneity and inadequate penetrance. We believe that association analysis is more suitable for the exploration of genetic factors in sporadic cases of microtia. Here we designed a panel that included all the published genes known to be associated with external/middle ear deformity or inner ear deformity in microtia-atresia patients. Ultimately, there were 65 rare mutations in 42 genes identified in 27 patients. For the low-frequency and common variants, the association studies were utilized to explore candidate genes for microtia-atresia and identified several candidate genes which may cause severe microtia-atresia.

In our exploration of rare mutations that have been reported to be pathogenic in HGMD, we found seven mutations what have been reported of which two mutations, one in *TBX1* (c.928G > A, p.G310S) and one in *TWIST1* (c.283_285dupAGC, p.S95dup), were likely responsible for the severe microtia-atresia. The individuals carrying these two mutations presented with external ear malformations. *TBX1* is a T-box transcription factor that maps to the center of the DiGeorge syndrome (DGS) chromosomal region on 22q11.2. A haploinsufficiency of the *TBX1* gene is responsible for most of the physical malformations of DGS, including deformed ears, which are typically low set and deficient in the vertical diameter with abnormal folding of the pinna; patients with DGS also exhibit upward and downward slanting eyes, short philtrum, relatively small mouth, cardiac malformation, and micrognathia [30, 31]. Nonsyndromic craniosynostosis-1 (CRS1) is caused by heterozygous mutations in *TWIST1* gene on chromosome 7p21. *TWIST1* belongs to the basic helix-loop-helix (bHLH) class of transcriptional regulators that recognize a consensus DNA element called the E box [29]. CRS1 is also reported to present with external ear malformations. Seto et al. (2007) performed a mutation analysis in 164 infants with nonsyndromic single-suture craniosynostosis and identified novel heterozygous missense mutations in the *TWIST1* gene in two patients. In addition to craniosynostosis, one of the patients also presented with prominent horizontal crura of the ears and the other had small, square-shaped ears, a feature shared by his otherwise unaffected father, who also carried the mutation [35]. These observations suggest that *TBX1* and *TWIST1* may be causative genes for severe microtia-atresia.

In addition to the rare mutations exploration, the low-frequency and common variants were also explored to identify the potential pathogenic genes utilizing the associated studies. Genome-wide association studies (GWAS) to date have generally been driven by the hypothesis that a common variant leads to a common disease. However, the contribution of rare and low-frequency variants to human traits has been largely unexplored [36, 37]. Since rare variants are incompletely represented in GWAS, and custom genotyping arrays and imputation is poor with current reference panels, we only performed association studies of common and low-frequency genetic variants. For low-frequency variant burden testing, these studies demonstrated a significant association with *PLEC*, *USH2A*, *FREM2*, *DCHS1*, *GLI3* and *POMT1*. For common variants, we identified associations of *DCHS1* (lead SNP, rs12288387; *P* = 1.869 × 10^− 45^) and *GBA* (lead SNP, rs144123706; *P* = 1.054 × 10^− 33^) with severe microtia-atresia. These findings provide evidence that a well-designed GWAS can identify new microtia-associated genes.

Association studies identified nineteen rare mutations in seven candidate genes. Both low-frequency and common-variants association studies strongly suggested that *DCHS1* was a cause of microtia-atresia. More importantly, the protein protocadherin-16, encoded by *DCHS1* involved in the Wnt (wingless/INT) signaling pathway, which, together with bone morphogenetic proteins (Bmps), fibroblast growth factors (Fgfs) and retinoic acid, is involved in external ear development [9, 38]. Dysregulation of these signaling pathways, triggered by genetic or environmental factors, constitutes a potential source of malformation. Members of the Wnt family have been implicated in the formation of NCCs and in external ear development. The protein protocadherin-16 encoded by *DCHS1* is a member of the cadherin superfamily whose spatial and temporal expression is critical to the formation of the neural crest [39]. *Dchs1*–*Fat4* has been reported to regulate the behavior of polarized cells during skeletal morphogenesis [40]. Thus, our findings provide further evidence that *DCHS1* is strongly associated with the pathogenesis of severe microtia-atresia. The rare p.R161C mutation in *DCHS1*, identified in one individual, predicted to be deleterious by different methods was also strongly suggested to cause severe microtia-atresia.

Of the other eighteen rare mutations identified in seven candidate genes, three mutations in two genes, *USH2A* and *FRME2*, were predicted to be deleterious. The protein usherin encoded by *USH2A* is found in the basement membrane, and may be important in the development and homeostasis of the inner ear and retina. Mutations within this gene have been associated with Usher syndrome type IIa and retinitis pigmentosa [41]. The other protein encoded by *FREM2* is also an integral membrane protein and localizes to the basement membrane. The protein is important for the integrity of skin and renal epithelia. Mutations in this gene are associated with Fraser syndrome, which is characterized by cryptophthalmos, syndactyly, and abnormalities of the respiratory and urogenital tracts [42]. However, none of these conditions has been associated with external ear deformity. The relationship between these two genes and microtia-atresia requires further confirmation.

Previous studies have provided compelling evidence that *HOXA2* mutations cause a potential discrete form of severe microtia-atresia. Both heterozygous and homozygous alleles of *HOXA2* have been identified in three family studies [18, 33, 34]. In affected individuals, ear morphogenesis is bilaterally small and characterized by hypoplastic pinnae with a thick, over-folded helix. Additional phenotypes include external auditory canal hypoplastic, middle and inner ear malformations, and incomplete cleft palate. The clinical features of individuals with a homozygous mutation of *HOXA2* are much more severe than those with a heterozygous mutation. Inactivation of *Hox2* in a mouse model at early stages of development leads to the absence of the pinna, whereas its late inactivation results in a hypomorphic auricle [43]. However, both the current study and previous studies attempted to identify *HOXA2* mutations in severe microtia-atresia patients by sequencing, but failed to find any potential causative mutations that might underlie a subgroup of well-defined microtia-atresia cases [18].

## Conclusion

Our results significantly improved our understanding of the genetic pathogenesis of severe microtia-atresia with regards to the rare, low-frequency, and common variants of detected genes. We identified several genes especially *DCHS1* that strongly be associated with microtia-atresia through low-frequency or common variants association study. A rare mutation (c.481C > T, p.R161C) in *DCHS1* identified in one individual may be deleterious and may cause microtia-atresia. The findings provide new insights into genetic exploration of external ear deformities.

## Additional files

Additional file 1: Summary of the 131 targeted genes. The “Gene symbol”, “Accession number”, “Size of gene (bp)”, “Size of gene exons (bp)”, “Inheritance”, “Human syndrome” were included in the table. (DOCX 33 kb)

Additional file 2: Rare or novel mutations identified in severe microtia-astresia patients. Rare mutations were filtered with a minor allele frequency < 0.5% in the 1000 Genomes (1KG) Project, and < 1% in Exome Sequencing Project (ESP6500) and the Genome Aggregation Database (gnomAD). (DOCX 36 kb)

Additional file 3: **Figure S1.** Principle component analysis (PCA) used to stratify the population. Results are based on data from 40 patients and 208 controls from the 1KG Project. (TIF 207 kb)

Additional file 4: Individual variant-association test results. For the common variants ((MAF ≥ 0.01 in the 1KG Project)), the Plink software was used to search for single variants that might increase microtia-atresia risk. The Bonferroni-corrected significance level (*P* = 4.95 × 10^− 5^) was used to identify the significant variants. (XLSX 113 kb)

Additional file 5: Results of gene-based, low-frequency variant association tests. For the low-frequency variants (MAF < 0.01 in the 1KG Project), the SKAT-O method was used to explore whether the burden of these variants in any of the tested genes was higher in microtia patients compared with controls, with significance level = 7.81 × 10^− 4^. (DOCX 20 kb)

Additional file 6: **Figure S2.** Conservation and functional analysis of the p.I4386T mutation in *USH2A***.** (A) Conservation of the Ile4386 residue of usherin protein. (B) Predicted secondary structures of the wild and mutant protein sequences flanking the mutations. The diagrams show the protein sequences with their secondary structures and their confidence values at the aligned positions. The secondary structure is annotated as follows: pink cylinder (alpha-helix); yellow arrow (beta-sheet); black line (coil); Conf, confidence; Pred, predict; H in Pred line (Helix); C in Pred line (coil); E in Pred line (sheet); AA, amino acid;↑, mutant amino acid. (TIF 677 kb)

Additional file 7: **Figure S3.** Predicted secondary structures of the wild and mutant protein sequences flanking the remaining candidate rare mutations. The diagrams show the protein sequences with their secondary structures and their confidence values at the aligned positions. The secondary structure is annotated as follows: pink cylinder (alpha-helix); yellow arrow (beta-sheet); black line (coil); Conf, confidence; Pred, predict; H in Pred line (Helix); C in Pred line (coil); E in Pred line (sheet); AA, amino acid;↑, mutant amino acid. (PDF 1140 kb)

## Abbreviations

1KG Project: 1000 Genomes Project
DGS: DiGeorge syndrome
GWAS: Genome-wide association study
HGMD: Human Gene Mutation Database
MAF: Minor allele frequency
NGS: Next-generation sequencing
