# Supplementary material for: Identification of sequence variants associated with severe microtia-astresia by targeted sequencing
Source: BMC Med Genomics. 2019 Jan 28;12:28. doi: 10.1186/s12920-019-0475-x (PMC6348636; doi:10.1186/s12920-019-0475-x)
Supplement: Supplementary file 1 — Summary of the 131 targeted genes. The “Gene symbol”, “Accession number”, “Size of gene (bp)”, “Size of gene exons (bp)”, “Inheritance”, “Human syndrome” were included in the table. (DOCX 33 kb) [file 12920_2019_475_MOESM1_ESM.docx]

**Additional file 1.** Summary of the 131 targeted genes

| **Gene symbol** | **Accession** | **Size of gene (bp)** | **Size of gene exons (bp)** | **Inheritance** | **Human syndrome** |
| --- | --- | --- | --- | --- | --- |
| *ABHD5* | NM_016006 | 31843 | 5346 | AR | Triglyceride storage disease with ichthyosis |
| *ADGRV1/GPR98* | XM_017009969 | 605637 | 19113 | N/A | Usher syndrome 2 and familial febrile seizures |
| *AMER1* | NM_152424 | 20628 | 8441 | XD | Osteopathia striata with cranial sclerosis |
| *ATRX* | NM_138270 | 281400 | 11185 | XR | Mental retardationN/Ahypotonic facies syndrome XN/Alinked, 1 |
| *B3GALTL* | NM_194318 | 132300 | 4198 | AR | Peters plus syndrome  MULTIPLE JOINT DISLOCATIONS, SHORT STATURE, CRANIOFACIAL DYSMORPHISM, AND CONGENITAL HEART DEFECTS |
| *B3GAT3* | NM_001288722 | 6880 | 4057 | AR |  |
| *BMP4* | NM_130850 | 7100 | 2145 | AD | Microphthalmia syndromic 6 (MCOPS6) |
| *BMP5* | NM_021073 | 120138 | 2163 | N/A | Keshan Disease and Epileptic Encephalopathy, ChildhoodN/AOnset |
| *CDC6* | NM_001254 | 15268 | 3041 | AR | MeierN/AGorlin syndrome 5 |
| *CDT1* | NM_030928 | 5481 | 2665 | AR | MeierN/AGorlin syndrome 4 |
| *CHD7* | NM_017780 | 189263 | 11542 | AD/Sporadic | CHARGE association |
| *CHST3* | NM_004273 | 49203 | 6970 | AR | Multiple joint dislocations, short stature, craniofacial dysmorphism, and congenital heart defects |
| *CHUK* | NM_001278 | 41221 | 3503 | AR | Cocoon syndrome |
| *CIB2* | NM_001271888 | 26930 | 1583 | AR | Usher syndrome, type 1J (USH1J) |
| *CLPP* | NM_006012 | 7453 | 1145 | AR | Deafness, autosomal recessive 81 (PRLTS3) |
| *CLRN1* | NM_001256819 | 46433 | 3976 | AR | Usher syndrome, type 3 (USH3) |
| *COL11A1* | NM_080629 | 232030 | 7376 | AD/AR | Marshall syndrome (MRSHS) |
| *COL2A1* | NM_033150 | 31538 | 5033 | AD/AR | Stickler syndrome type 1 (STL1) |
| *COL4A3* | NM_000091 | 150228 | 8045 | AR | Alport syndrome, autosomal recessive |
| *COL4A4* | NM_000092 | 161849 | 10293 | AR | Alport syndrome, autosomal recessive |
| *COL4A5* | NM_033380 | 257702 | 6779 | XR | Alport syndrome, XN/Alinked recessive (ATS) |
| *COL9A1* | NM_001851 | 87044 | 3971 | AR | Stickler syndrome, type 4 (STL4) |
| *CYP26B1* | NM_001277742 | 18625 | 4550 | AR | Radiohumeral fusions with other skeletal and craniofacial anomalies (RHFCA) |
| *DlX6* | NM_005222 | 5062 | 1892 | N/A | RappN/AHodgkin Syndrome and Split Foot |
| *DCHS1* | NM_003737 | 34526 | 10738 | AR | Van Maldergem Wetzburger Verloes syndrome |
| *DFNB31* | NM_001083885 | 101136 | 4327 | N/A | autosomal recessive nonN/Asyndromic deafness and Usher Syndrome |
| *DHODH* | NM_001361 | 16674 | 2418 | AR | Miller syndrome (POADS) |
| *DLK1* | NM_003836 | 8266 | 1585 | N/A | Uniparental disomy, paternal, chromosome 14 |
| *DLX5* | NM_005221 | 4442 | 1421 | AR | SplitN/Ahand/foot malformation 1 with sensorineural hearing loss (SHFM1D) |
| *EDN1* | NM_001168319 | 6899 | 2260 | AD | Question mark ears, isolated (QME) |
| *EDNRA* | NM_001256283 | 64038 | 4142 | AD | Migraine (MA) |
| *EDNRB* | NM_001122659 | 23351 | 6462 | AD/AR | Waardenburg syndrome type 4A (WS4A) |
| *EFTUD2* | NM_004247 | 49339 | 4567 | AR/AD | Growth and mental retardation, mandibulofacial dysostosis, microcephaly, and cleft palate |
| *EVC* | NM_153717 | 103108 | 6409 | AD | CurryN/AHall syndrome (WAD) |
| *EYA1* | NM_001288574 | 164800 | 4877 | AD | ANTERIOR SEGMENT ANOMALIES WITH OR WITHOUT CATARACT |
| *FAT4* | NM_001291285 | 176521 | 19371 | AR | VAN MALDERGEM SYNDROME 2 |
| *FGF10* | NM_004465 | 83688 | 624 | AD | LevyN/AHollister syndrome (LADD) |
| *FGF3* | NM_005247 | 9457 | 1545 | AR | Deafness with labyrinthine aplasia microtia and microdontia (LAMM) |
| *FGF8* | NM_001206389 | 10240 | 1250 | AD | Kallmann syndrome 6 (KAL6) |
| *FGFR2* | NR_073009 | 120129 | 7346 | AD | BLEPHAROPHIMOSIS, EPICANTHUS INVERSUS, AND PTOSIS 3, FORMERLY |
| *FKRP* | NM_024301 | 12530 | 3620 | AR | WalkerN/AWarburg congenital muscular dystrophy |
| *FKTN* | NM_006731 | 82989 | 8559 | AR | WalkerN/AWarburg congenital muscular dystrophy |
| *FOXI1* | NM_144769 | 3813 | 3685 | AR | Enlarged vestibular aqueduct syndrome (DFNB4) |
| *FRAS1* | NM_001166133 | 389064 | 16661 | AR | Cryptophthalmos syndrome |
| *FREM2* | NM_207361 | 200095 | 16138 | AR | Cryptophthalmos syndrome |
| *GABRD* | NM_000815 | 11425 | 1916 | N/A | Chromosome 1p36 deletion syndrome |
| *GATA1* | NM_002049 | 7736 | 1473 | Sporadic | Complete trisomy 21 syndrome |
| *GBA* | NM_001005741 | 10415 | 3027 | AR | Gaucher disease, perinatal lethal |
| *GDF6* | NM_001001557 | 18463 | 3699 | AD | KlippelN/AFeil syndrome 1, autosomal dominant (KFS1) |
| *GLI3* | NM_000168 | 276071 | 8199 | AD | PallisterN/AHall syndrome |
| *GRIP1* | NM_021150 | 331715 | 5036 | AR | Cryptophthalmos syndrome |
| *GSC* | NM_173849 | 1940 | 1186 | AR | Short stature, auditory canal atresia, mandibular hypoplasia, skeletal abnormalities (SAMS) |
| *HARS2* | NM_001278731 | 7893 | 2671 | AR | Perrault syndrome 2 (PRLTS2) |
| *HMX1* | NM_018942 | 4771 | 1894 | AR | Oculoauricular syndrome |
| *HOXA1* | NM_005522 | 3012 | 3086 | AR | Athabaskan brainstem dysgenesis (ABDS) |
| *HOXA2* | NM_006735 | 2422 | 1776 | AD/AR | Microtia, hearing impairment, and cleft palate |
| *HOXB6* | NM_018952 | 9236 | 1672 | AD/AR/XN/Alinked | [Hypospadias](http://www.malacards.org/card/hypospadias) |
| *HSD17B4* | NM_001292028 | 89839 | 3124 | AR | Gonadal dysgenesis with auditory dysfunction, autosomal recessive inheritance (PRLTS1) |
| *IRF6* | NM_001206696 | 20553 | 4496 | AD | Van der Woude syndrome (VWS1) |
| *ISPD* | NM_001101417 | 333796 | 5514 | AR | MUSCULAR DYSTROPHYN/ADYSTROGLYCANOPATHY (CONGENITAL WITH BRAIN AND EYE ANOMALIES), TYPE A, 7 |
| *KAT6B* | NM_001256469 | 206210 | 8918 | AD | Mental retardation, congenital heart disease, blepharophimosis, blepharoptosis and hypoplastic teeth |
| *KCNAB2* | NM_001199861 | 108896 | 5806 | N/A | Chromosome 1p36 deletion syndrome |
| *KCNJ10* | NM_002241 | 32795 | 5304 | AR | Enlarged vestibular aqueduct syndrome (DFNB4) |
| *KCTD1* | NM_001258222 | 202492 | 4219 | AD | Scalp ear nipple syndrome |
| *KDM6A* | NR_111960 | 239437 | 6019 | AD | Kabuki makeN/Aup syndrome (KABUK1) |
| *KMT2D* | NM_003482 | 36350 | 19365 | N/A | Kabuki makeN/Aup syndrome (KABUK1) |
| *LARGE* | NM_004737 | 647355 | 4185 | AR | WalkerN/AWarburg congenital muscular dystrophy |
| *LARS2* | NM_015340 | 160254 | 4181 | AR | Perrault syndrome 4 (PRLTS4) |
| *LMBRD1* | NM_018368 | 121409 | 2292 | AR | METHYLMALONIC ACIDURIA AND HOMOCYSTINURIA, cblF TYPE |
| *MBD5* | NM_018328 | 492465 | 6001 | AD | CHROMOSOME 2q23.1 DELETION SYNDROME |
| *MED12* | NM_005120 | 23899 | 6924 | XR | FG syndrome |
| *MEG3* | NR_046473 | 28198 | 11494 | N/A | Uniparental disomy, paternal, chromosome 14 |
| *MITF* | NM_001184967 | 204782 | 6195 | AD | Waardenburg syndrome type 2A (WS2A) |
| *NDP* | NM_000266 | 24898 | 1999 | XR | Atrophia bulborum hereditaria (ND) |
| *NIN* | NM_182946 | 105294 | 11018 | AR | SECKEL SYNDROME 7 |
| *NKX3N/A2* | NM_001189 | 3661 | 2239 | AR | SpondyloN/AmegaepiphysealN/Ametaphyseal dysplasia (SMMD) |
| *ORC1* | NM_001190819 | 31643 | 3428 | AR | MeierN/AGorlin syndrome |
| *ORC4* | NM_002552 | 90364 | 7496 | AR | MeierN/AGorlin syndrome 2 |
| *ORC6* | NR_037620 | 8749 | 1804 | AR | MeierN/AGorlin syndrome 3 |
| *OSR1* | NM_145260 | 7127 | 1891 | N/A | N/A |
| *PAM16* | NM_016069 | 11122 | 579 | AR | Chondrodysplasia, megarbaneN/AdagherN/Amelki type |
| *PAX3* | NM_001127366 | 99110 | 6132 | AD/AR | KleinN/AWaardenberg's syndrome (WS3) |
| *PAX8* | NM_013952 | 62925 | 4152 | N/A | Thyroid agenesis (CHNG2) |
| *PCDH15* | NM_001142769 | 992600 | 12842 | AR | Deafness, autosomal recessive 23 (DFNB23) |
| *PCNT* | NM_006031 | 121647 | 10513 | AR | Osteodysplastic primordial dwarfism, type 2 |
| *PDZD7* | NM_024895 | 13826 | 4402 | AR | Usher syndrome, type 2A (USH2A) |
| *PLEC* | NM_201383 | 27378 | 16423 | AR | Epidermolysis bullosa simplex with pyloric atresia |
| *POC1A* | NM_015426 | 79458 | 2340 | AR | SHORT STATURE, ONYCHODYSPLASIA, FACIAL DYSMORPHISM, AND HYPOTRICHOSIS SYNDROME |
| *POLR1C* | NM_203290 | 4470 | 1311 | AR | Mandibulofacial dysostosis, Treacher Collins type, autosomal recessive (TCS3) |
| *POLR1D* | NM_152705 | 45644 | 3015 | AD | Treacher Collins syndrome 2 (TCS2) |
| *POMT1* | NM_001077366 | 20905 | 3630 | AR | WalkerN/AWarburg congenital muscular dystrophy |
| *POMT2* | NM_013382 | 45927 | 4853 | AR | WalkerN/AWarburg congenital muscular dystrophy |
| *PRDM16* | NM_022114 | 369444 | 13593 | N/A | Chromosome 1p36 deletion syndrome |
| *PRKCZ* | NM_001242874 | 111410 | 3058 | N/A | Chromosome 1p36 deletion syndrome |
| *PRKRA* | NM_001139517 | 19344 | 2399 | AR | Dystonia 16 (DYT16) |
| *PRRX1* | NM_006902 | 75229 | 4066 | AR/AD | Dysgnathia complex (AGOTC) |
| *PRRX2* | NM_016307 | 57032 | 1305 | N/A | N/A |
| *RARA* | NM_001024809 | 15625 | 4350 | N/A | N/A |
| *RBM10* | NM_005676 | 41598 | 3833 | XR | TARP syndrome |
| *RNU4ATAC* | NR_023343 | 130 | 129 | AR | Osteodysplastic primordial dwarfism, type 1 |
| *RPS28* | NM_001031 | 897 | 378 | N/A | DiamondN/ABlackfan anemia with microtia and cleft palate |
| *RTL1* | NM_001134888 | 4193 | 4192 | N/A | Uniparental disomy, paternal, chromosome 14 |
| *SALL1* | NM_002968 | 15298 | 5356 | AD | Townes syndrome |
| *SEMA3E* | NM_012431 | 285258 | 6711 | AD/Sporadic | CHARGE association |
| *SF3B4* | NM_005850 | 4936 | 1987 | AD/AR | Nager syndrome (AFD1) |
| *SIX1* | NM_005982 | 4739 | 2685 | AD | MelnickN/AFraser syndrome |
| *SIX2* | NM_016932 | 4219 | 2152 | N/A | N/A |
| *SIX4* | NM_017420 | 14597 | 6272 | N/A | N/A |
| *SIX5* | NM_175875 | 4455 | 3328 | AD | Branchiootorenal syndrome 2 (BOR2) |
| *SKI* | NM_003036 | 81519 | 5700 | N/A | Chromosome 1p36 deletion syndrome |
| *SLC26A4* | NM_000441 | 57173 | 4907 | AR | Enlarged vestibular aqueduct syndrome (DFNB4) |
| *SLC2A9* | NM_001001290 | 214025 | 2065 | AD | Renal hypouricemia 2 (RHUC2) |
| *SMAD4* | NM_005359 | 54829 | 8759 | AD | Myhre syndrome |
| *SNAI2* | NM_003068 | 3761 | 2106 | AR | Waardenburg syndrome type 2D (WS2D) |
| *SOX8* | NM_014587 | 5172 | 3046 | AD | Chromosome 16N/Arelated alphaN/Athalassemia/mental retardation syndrome |
| *TBX1* | NM_080646 | 22843 | 2792 | AD | DiGeorge sequence (DGS) |
| *TBX15* | NM_152380 | 106514 | 3487 | AR | Cousin syndrome |
| *TCOF1* | NM_001008657 | 26994 | 6048 | N/A | Treacher collins syndrome 1 (TCS1) |
| *TFAP2A* | NM_001042425 | 22882 | 4243 | AD | Branchiooculofacial syndrome |
| *TP63* | NM_003722 | 265853 | 6508 | AD | Ectrodactyly, ectodermal dysplasia, and cleft lip/palate syndrome 3 |
| *TRPS1* | NM_001282902 | 259516 | 10291 | AD | Trichorhinophalangeal syndrome type 3 (TRPS3) |
| *TTC37* | NM_014639 | 91111 | 5661 | AR | Trichohepatoenteric syndrome |
| *TWIST1* | NM_000474 | 2205 | 1664 | AD | BLEPHAROPHIMOSIS, EPICANTHUS INVERSUS, AND PTOSIS 3, FORMERLY |
| *TWSG1* | NM_020648 | 67654 | 3751 | N/A | N/A |
| *USH1C* | NM_001297764 | 50522 | 3292 | AR | Deafness, autosomal recessive 18 (DFNB18A) |
| *USH1G* | NM_001282489 | 7183 | 4729 | N/A | Usher syndrome type 1G (USH1G). |
| *USH2A* | NM_206933 | 800503 | 20343 | AR | Usher syndrome, type 2A (USH2A) |
| *VRK1* | NM_003384 | 84268 | 1690 | AR | Pontocerebellar hypoplasia type 1 (PCH1A) |
| *WFS1* | NM_006005 | 33416 | 3864 | AR | Diabetes mellitus AND insipidus with optic atrophy AND deafness (WFS1) |
| *WNT3* | NM_030753 | 56255 | 3350 | AR | Tetraamelia, autosomal recessive |
| *WNT5A* | NM_001256105 | 15684 | 6239 | AR/AD | Robinow syndrome (DRS) |

AR: Autosomal Recessive; AD: Autosomal Dominant; N/A: Not applicable.
