# Supplementary material for: Identification of sequence variants associated with severe microtia-astresia by targeted sequencing
Source: BMC Med Genomics. 2019 Jan 28;12:28. doi: 10.1186/s12920-019-0475-x (PMC6348636; doi:10.1186/s12920-019-0475-x)
Supplement: Supplementary file 2 — Rare or novel mutations identified in severe microtia-astresia patients. Rare mutations were filtered with a minor allele frequency < 0.5% in the 1000 Genomes (1KG) Project, and < 1% in Exome Sequencing Project (ESP6500) and the Genome Aggregation Database (gnomAD). (DOCX 36 kb) [file 12920_2019_475_MOESM2_ESM.docx]

**Additional file 2.** Rare or novel mutations identified in severe microtia-astresia microtia patients

| **Sample ID** | **Gene Symbol** | **Consequence** | **GenBank accession No.** | **Exon** | **Mutation** | **PopFreqMax in 1000GM, ESP6500 or gnomAD** | **SIFT**  **_score** | **SIFT**  **_Pred** | **Polyphen2_HDIV_score** | **Polyphen2_HDIV_pred** | **Phylop in vertebrate** | **HGMD** |
| --- | --- | --- | --- | --- | --- | --- | --- | --- | --- | --- | --- | --- |
| 1454 | *LMBRD1* | nonsynonymous | NM_018368 | exon15 | c.1435C>T, p.R479W | 0.00002508 | 0.001 | D | 1 | D | 0.045 | NR |
|  | *PLEC* | nonsynonymous | NM_201378 | exon31 | c.5273C>T, p.A1758V | 0.0001578 | 0.535 | T | 1 | D | 0.897 | NR |
|  | *KMT2D* | nonsynonymous | NM_003482 | exon34 | c. 9326C>A, p.P3109H | 0.00004726 | 0.001 | D | 0.214 | B | 0.63 | NR |
|  | *RARG* | nonsynonymous | NM_001243732 | exon5 | c. 949G>A, p.G317R | 0.000008475 | 0.001 | D | 1 | D | 0.871 | NR |
| 1467 | *PLEC* | nonsynonymous | NM_201378 | exon32 | c.11563G>A, p.G3855S | 0.00002367 | 0.652 | T | 0 | B | -2.741 | NR |
|  | *USH2A* | nonsynonymous | NM_007123 | exon6 | c.1007A>G, p.N336S | . | 0.048 | D | 0.218 | B | 0.991 | NR |
| 1549 | *POMT2* | nonsynonymous | NM_013382 | exon16 | c.1714A>G, p.I572V | 0.000033 | 0.025 | D | 0.061 | B | 0.899 | NR |
|  | *LMBRD1* | nonsynonymous | NM_018368 | exon1 | c.14G>C, p.G5A | . | 1 | T | 0.999 | D | 0.666 | NR |
| 1552 | **GBA* | nonsynonymous | NM_001005741 | Exon10 | c. 1292A>G, p.N431S | 0.00001657 | 0.169 | T | 0.962 | D | 0.991 | DM？ |
| 1575 | *USH2A* | nonsynonymous | NM_206933 | exon27 | c. 5389A>G, p.N1797D | . | 1 | T | 0 | B | -0.068 | NR |
| 1605 | *KMT2D* | nonsynonymous | NM_003482 | exon31 | c. 6578C>T, p.P2193L | 0.00008092 | 0.204 | T | 0.241 | B | -0.045 | NR |
| 1608 | *DFNB59* | nonsynonymous | NM_001042702 | exon2 | c. 73C>T, p.L25F | . | 0.003 | D | 1 | D | 0.871 | NR |
|  | *FAT4* | nonsynonymous | NM_001291285 | exon1 | c. 3982A>C, p.M1328L | 0.000008281 | 0.125 | T | 0.905 | P | 1.062 | NR |
|  | *GDF6* | nonsynonymous | NM_001001557 | exon2 | c. 701G>T, p.R234L | . | 0.013 | D | 0.215 | B | 0.853 | NR |
| 1611 | *POMT2* | nonsynonymous | NM_013382 | exon21 | c. 2188C>T, p.P730S | 0.000008247 | 0.093 | T | 0.616 | P | 0.83 | NR |
|  | *ORC1* | nonsynonymous | NM_001190818 | exon9 | c. 1411G>T, p.A471S | 0.00004179 | 0.019 | D | 0 | B | 0.084 | NR |
|  | *FRAS1* | nonsynonymous | NM_001166133 | exon23 | c. 2756A>C, p.Q919P | . | 0.304 | T | 0 | B | 0.02 | NR |
|  | *FAT4* | nonsynonymous | NM_001291285 | exon1 | c. 3371C>T, p.A1124V | . | 0 | D | 0.999 | D | 0.871 | NR |
| 1614 | *GRIP1* | nonsynonymous | NM_001178074 | exon21 | c. 2788C>G, p.P930A | . | 0.113 | T | 0.915 | P | 0.917 | NR |
|  | *GLIS2* | nonsynonymous | NM_032575 | exon6 | c. 1153G>C, p.A385P | . | 0.002 | D | 0.001 | B | 0.917 | NR |
| 1633 | *SFXN3* | nonsynonymous | NM_030971 | exon5 | c. 437C>T, p.T146I | . | 0.003 | D | 0.859 | P | 0.871 | NR |
|  | *SIX5* | nonsynonymous | NM_175875 | exon2 | c. 1193C>T, p.A398V | . | 0 | D | 0.19 | B | 0.917 | NR |
| 1653 | *HOXA3* | nonsynonymous | NM_030661 | exon2 | c. 263C>T, p.P88L | . | 0.023 | D | 0.002 | B | 0.917 | NR |
| 1742 | **TBX1* | nonsynonymous | NM_080646 | exon8 | c. 929G>C, p.G310A c.C11123T, p.P3708L | 0.00008616 | 0.087 | T | 0.826 | P | 0.897 | DM |
|  | *USH2A* | nonsynonymous | NM_206933 | exon57 | c.11123C>T, p.P3708L | . | 0.223 | T | 1 | D | 0.917 | NR |
| 1782 | *SIX4* | nonsynonymous | NM_017420 | exon1 | c. 694G>C, p.E232Q | 0.000008387 | 0.001 | D | 1 | D | 0.871 | NR |
| 1783 | *TP63* | nonsynonymous | NM_001114980 | exon12 | c. 1703A>G, p.N568S | 0.0000165 | 0.178 | T | 0.002 | B | 1.062 | NR |
|  | *ATRX* | nonsynonymous | NM_138270 | exon8 | c. 3089C>T, p.T1030I | . | 0.011 | D | 0.028 | B | -0.033 | NR |
| XH1790 | *DCHS1* | nonsynonymous | NM_003737 | exon2 | c. 481C>T, p.R161C | 0.00003335 | 0.069 | T | 1 | D | -0.001 | NR |
| XH1493 | **SLC26A4* | nonsynonymous | NM_000441 | exon6 | c. 757A>G, p.I253V | 0.0006933 | 0.482 | T | 0.877 | P | 1.062 | DM |
| XH1528 | *KANK3* | nonsynonymous | NM_198471 | exon3 | c. 870G>T, p.E290D | . | 0.504 | T | 0.018 | B | -0.041 | NR |
| XH1531 | *KMT2D* | nonframeshift | NM_003482 | exon39 | c.11758_11759insAGCAGCAGC, p.L3920insQQQL | . | . | . | . | . | . | NR |
|  | *TTC37* | stopgain | NM_014639 | exon21 | c. 2151G>A, p.W717X | . | . | . | . | . | 0.871 | NR |
|  | *B3GLCT* | nonsynonymous | NM_194318 | exon15 | c. 1380A>G, p.I460M | . | 0.002 | D | 0.989 | D | 1.062 | NR |
|  | *PCNT* | nonsynonymous | NM_001315529 | exon28 | c. 5490C>G, p.D1830E | . | 0.11 | T | 0.999 | D | 0.033 | NR |
| XH1535 | *POMT1* | nonsynonymous | NM_001136114 | exon16 | c. 1428G>C, p.L476F | 0.0001156 | 0.556 | T | 0.001 | B | -0.043 | NR |
| XH1546 | *KAT6B* | nonframeshift | NM_001256468 | exon18 | c.3559_3561del, p.1187_1187del | 0.001196 | . | . | . | . | . | NR |
|  | *USH2A* | nonsynonymous | NM_206933 | exon42 | c. 8420C>T, p.T2807I | . | 0.065 | T | 0.998 | D | 0.917 | NR |
|  | *POMT1* | nonsynonymous | NM_001136114 | exon16 | c. 1428G>C, p.L476F | 0.0001156 | 0.556 | T | 0.001 | B | -0.043 | NR |
| XH1559 | *PAX8* | nonsynonymous | NM_003466 | exon5 | c. 473C>T, p.T158M | 0.0001159 | 0.003 | D | 1 | D | 0.913 | NR |
|  | *KMT2D* | nonsynonymous | NM_003482 | exon17 | c. 4682T>G, p.V1561G | 0.001047 | 0.134 | T | 0.31 | B | 1.062 | NR |
| XH1563 | *ISPD* | nonframeshift | NM_001101417 | exon1 | c.21_44del, p.7_15del | . | . | . | . | . | . | NR |
|  | *GLI3* | nonframeshift | NM_000168 | exon15 | c. 3107C>T, p.A1036V | 0.00007006 | 0.016 | D | 0 | B | 0.897 | NR |
| XH1566 | *USH2A* | nonsynonymous | NM_206933 | exon55 | c.10871A>G, p.Q3624R | . | 0.271 | T | 0.012 | B | 0.079 | NR |
|  | *HMX1* | nonsynonymous | NM_018942 | exon2 | c. 946C>A, p.P316T | . | 0.005 | D | 0.999 | D | 0.893 | NR |
|  | *FKTN* | nonsynonymous | NM_006731 | exon4 | c. 184A>T, p.I62F | . | 0.042 | D | 0.589 | P | 0.02 | NR |
|  | *SLC1A5* | nonsynonymous | NM_005628 | exon1 | c. 544G>A, p.D182N | . | 0.013 | D | 1 | D | 0.871 | NR |
| XH1572 | *LARS2* | nonsynonymous | NM_015340 | exon21 | c. 2464C>T, p.L822F | . | 0.058 | T | 0.68 | P | 0.827 | NR |
|  | *TP63* | nonsynonymous | NM_001114980 | exon12 | c. 1505C>T, p.A502V | 0.00006056 | 1 | T | 0.124 | B | 0.871 | NR |
| XH1579 | **USH2A* | nonsynonymous | NM_206933 | exon63 | c.13157T>C, p.I4386T | . | 0.006 | D | 0.978 | D | 1.062 | DM |
|  | *USH2A* | nonsynonymous | NM_206933 | exon56 | c.10958A>G, p.N3653S | . | 0.409 | T | 0.489 | P | 0.991 | NR |
|  | *USH2A* | nonsynonymous | NM_007123 | exon6 | c. 1105G>A, p.V369M | . | 0.015 | D | 0.992 | D | 0.871 | NR |
|  | *COL4A3* | nonsynonymous | NM_000091 | exon28 | c. 2054C>T, p.P685L | 0.00003906 | 0.055 | T | 0.197 | B | 0.871 | NR |
|  | *FRAS1* | nonsynonymous | NM_025074 | exon74 | c.11911T>C, p.C3971R | . | . | . | 1 | D | 0.991 | NR |
| XH1582 | **TWIST1* | nonframeshift | NM_000474 | exon1 | c.283_285dupAGC, p.S95dup | . | . | . | . | . | . | DM? |
|  | *POMT1* | stopgain | NM_001136114 | exon18 | c.1790delG, p.W597X | 0.000008708 | . | . | . | . | . | NR |
|  | *PLEC* | nonsynonymous | NM_000445 | exon2 | c. 115A>G, p.S39G | 0.001536 | 0.381 | T | 0.002 | B | 0.991 | NR |
| XH1587 | *LMBRD1* | nonsynonymous | NM_018368 | exon16 | c. 1537T>C, p.C513R | . | 0.119 | T | 0.99 | D | 1.062 | NR |
| XH1594 | **USH2A* | nonsynonymous | NM_206933 | exon63 | c.12344G>A, p.R4115H | 0.0005825 | 0.072 | T | 0.462 | P | 0.871 | DM |
|  | *GLI3* | nonsynonymous | NM_000168 | exon7 | c. 857C>T, p.A286V | . | 0.18 | T | 0.024 | B | 0.917 | NR |
| XH1745 | **FKTN* | nonsynonymous | NM_006731 | exon5 | c. 556C>T, p.H186Y | 0.0002312 | 0.125 | T | 0.998 | D | 0.871 | DM |
|  | *MED12* | nonsynonymous | NM_005120 | exon15 | c. 2203G>A, p.A735T | 0.00002303 | 0.715 | T | 0.814 | P | 0.917 | NR |
| XH1748 | *OTOG* | nonsynonymous | NM_001277269 | exon18 | c. 2181C>G, p.F727L | . | 0.011 | D | . | . | -0.379 | NR |
| XH1784 | *FKTN* | stopgain | NM_001198963 | exon12 | c. 1282C>T, p.Q428X | . | . | . | . | . | 0.598 | NR |
| XH1785 | *CYP26B1* | nonsynonymous | NM_001277742 | exon2 | c. 280C>G, p.L94V | . | 0.837 | T | 0.384 | B | 0.917 | NR |
|  | *FREM2* | nonsynonymous | NM_207361 | exon22 | c. 8689C>T, p.R2897C | . | 0 | D | 1 | D | 0.871 | NR |

**Notes**: *indicates the mutation has been reported in HGMD database.

**Abbreviations:** SIFT: D = Deleterious, T = Tolerated; PolyPhen 2: D = Probably damaging, B = Benign, P = Possibly damaging; NR = not reported; DM: "disease causing" mutation.
