# Supplementary figures and images for: Identification of sequence variants associated with severe microtia-astresia by targeted sequencing
Source: BMC Med Genomics. 2019 Jan 28;12:28. doi: 10.1186/s12920-019-0475-x (PMC6348636; doi:10.1186/s12920-019-0475-x)

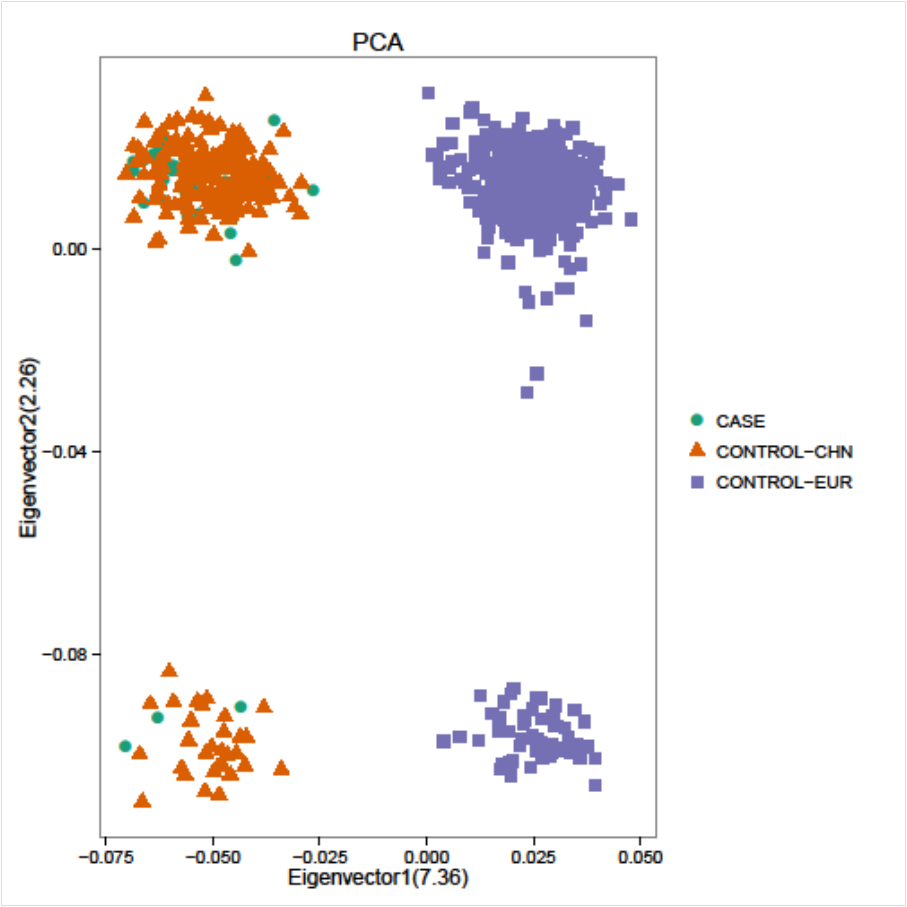

Supplement: Supplementary file 3 — Figure S1. Principle component analysis (PCA) used to stratify the population. Results are based on data from 40 patients and 208 controls from the 1KG Project. (TIF 207 kb) [file 12920_2019_475_MOESM3_ESM.tif]

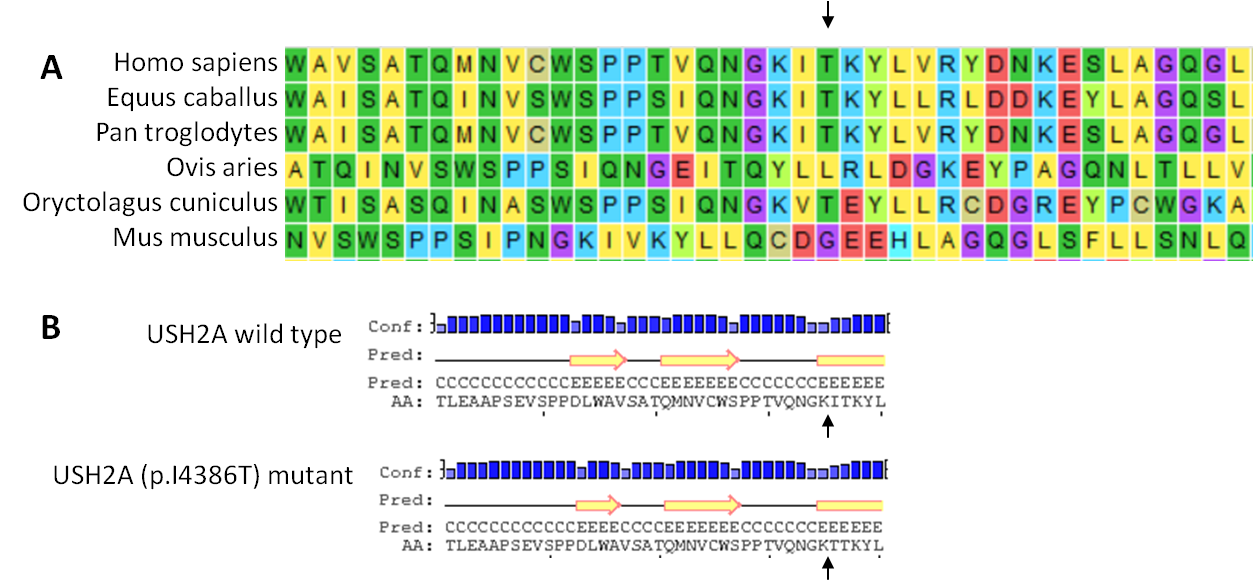

Supplement: Supplementary file 6 — Figure S2. Conservation and functional analysis of the p.I4386T mutation in USH2A. (A) Conservation of the Ile4386 residue of usherin protein. (B) Predicted secondary structures of the wild and mutant protein sequences flanking the mutations. The diagrams show the protein sequences with their secondary structures and their confidence values at the aligned positions. The secondary structure is annotated as follows: pink cylinder (alpha-helix); yellow arrow (beta-sheet); black line (coil); Conf, confidence; Pred, predict; H in Pred line (Helix); C in Pred line (coil); E in Pred line (sheet); AA, amino acid;↑, mutant amino acid. (TIF 677 kb) [file 12920_2019_475_MOESM6_ESM.tif]
