# Supplementary material for: Identification of sequence variants associated with severe microtia-astresia by targeted sequencing
Source: BMC Med Genomics. 2019 Jan 28;12:28. doi: 10.1186/s12920-019-0475-x (PMC6348636; doi:10.1186/s12920-019-0475-x)
Supplement: Supplementary file 5 — Results of gene-based, low-frequency variant association tests. For the low-frequency variants (MAF < 0.01 in the 1KG Project), the SKAT-O method was used to explore whether the burden of these variants in any of the tested genes was higher in microtia patients compared with controls, with significance level = 7.81 × 10− 4. (DOCX 20 kb) [file 12920_2019_475_MOESM5_ESM.docx]

**Additional file 5.** Results of gene-based, low-frequency variant association tests (significance level *P*= 7.81 × 10^-4^).

| Gene | Markers | *P*-value |
| --- | --- | --- |
| *PLEC* | 12 | 1.40 × 10^-10^ |
| *USH2A* | 16 | 1.11 × 10^-08^ |
| *FREM2* | 6 | 4.38 × 10^-05^ |
| *DCHS1* | 6 | 0.000288 |
| *GLI3* | 5 | 0.000297 |
| *POMT1* | 2 | 0.000297 |
| *CDC6* | 2 | 0.001967 |
| *FKTN* | 3 | 0.001967 |
| *LMBRD1* | 3 | 0.001967 |
| *SLC26A4* | 2 | 0.001967 |
| *NIN* | 3 | 0.002188 |
| *KMT2D* | 5 | 0.003996 |
| *FRAS1* | 7 | 0.004171 |
| *PCDH15* | 5 | 0.004896 |
| *FAT4* | 5 | 0.005717 |
| *COL4A3* | 2 | 0.012733 |
| *FOXI1* | 2 | 0.012733 |
| *GBA* | 2 | 0.012733 |
| *GDF6* | 2 | 0.012733 |
| *KAT6B* | 1 | 0.012733 |
| *MED12* | 2 | 0.012733 |
| *ORC1* | 2 | 0.012733 |
| *PCNT* | 2 | 0.012733 |
| *POMT2* | 2 | 0.012733 |
| *SEMA3E* | 1 | 0.012733 |
| *TP63* | 2 | 0.012733 |
| *TTC37* | 4 | 0.017107 |
| *SIX4* | 2 | 0.022453 |
| *COL4A4* | 2 | 0.025467 |
| *TWIST1* | 1 | 0.025467 |
| *TRPS1* | 2 | 0.047 |
| *PRDM16* | 3 | 0.051653 |
| *ABHD5* | 2 | 0.067181 |
| *ATRX* | 1 | 0.080645 |
| *CLPP* | 1 | 0.080645 |
| *CYP26B1* | 1 | 0.080645 |
| *GATA1* | 1 | 0.080645 |
| *GRIP1* | 1 | 0.080645 |
| *HMX1* | 1 | 0.080645 |
| *ISPD* | 1 | 0.080645 |
| *LARS2* | 1 | 0.080645 |
| *MITF* | 1 | 0.080645 |
| *PAX3* | 1 | 0.080645 |
| *PAX8* | 1 | 0.080645 |
| *SALL1* | 2 | 0.080645 |
| *SIX2* | 1 | 0.080645 |
| *SIX5* | 1 | 0.080645 |
| *SLC2A9* | 1 | 0.080645 |
| *TBX1* | 1 | 0.080645 |
| *USH1G* | 1 | 0.080645 |
| *WFS1* | 2 | 0.080645 |
| *COL11A1* | 2 | 0.138472 |
| *MBD5* | 2 | 0.153832 |
| *PDZD7* | 2 | 0.153832 |
| *DFNB31* | 3 | 0.16129 |
| *EYA1* | 1 | 0.16129 |
| *GABRD* | 1 | 0.16129 |
| *SOX8* | 1 | 0.16129 |
| *HSD17B4* | 2 | 0.191031 |
| *RTL1* | 1 | 0.239969 |
| *HOXA1* | 1 | 0.807805 |
| *SKI* | 1 | 0.807805 |
| *CDT1* | 1 | 1 |
| *DLK1* | 1 | 1 |
