# Supplementary material for: Identification of sequence variants associated with severe microtia-astresia by targeted sequencing
Source: BMC Med Genomics. 2019 Jan 28;12:28. doi: 10.1186/s12920-019-0475-x (PMC6348636; doi:10.1186/s12920-019-0475-x)
Supplement: Supplementary file 7 — Figure S3. Predicted secondary structures of the wild and mutant protein sequences flanking the remaining candidate rare mutations. The diagrams show the protein sequences with their secondary structures and their confidence values at the aligned positions. The secondary structure is annotated as follows: pink cylinder (alpha-helix); yellow arrow (beta-sheet); black line (coil); Conf, confidence; Pred, predict; H in Pred line (Helix); C in Pred line (coil); E in Pred line (sheet); AA, amino acid;↑, mutant amino acid. (PDF 1140 kb) [file 12920_2019_475_MOESM7_ESM.pdf]

Supplementary Fig. 3 Predicted secondary structures of the wild and mutant protein sequences flanking the remaining candidate rare mutations. The diagrams show the protein sequences with their secondary structures and their confidence values at the aligned positions. The secondary structure is annotated as follows: pink cylinder (alpha-helix); yellow arrow (beta-sheet); black line (coil); Conf, confidence; Pred, predict; H in Pred line (Helix); C in Pred line (coil); E in Pred line (sheet); AA, amino acid; ↑, mutant amino acid.

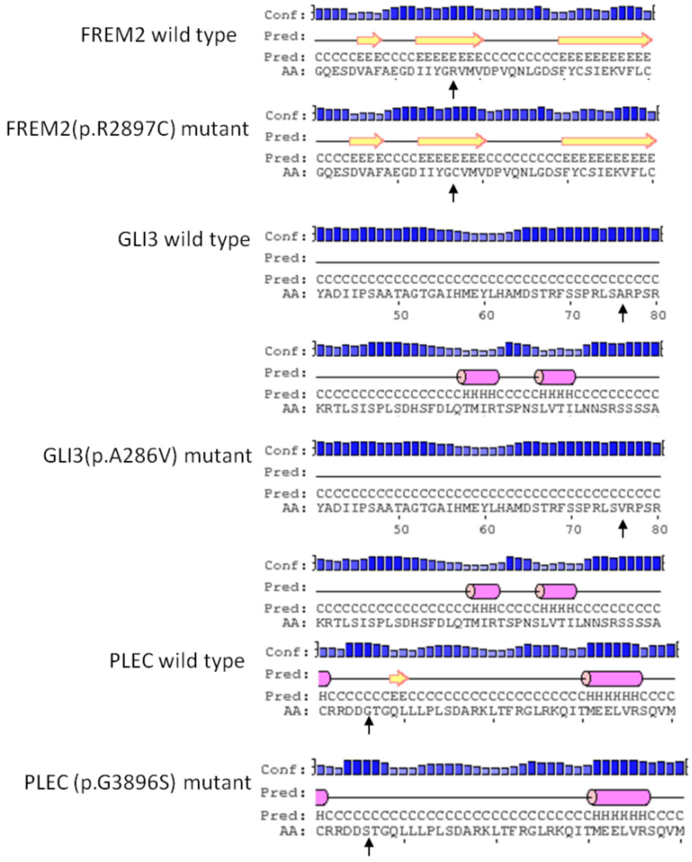

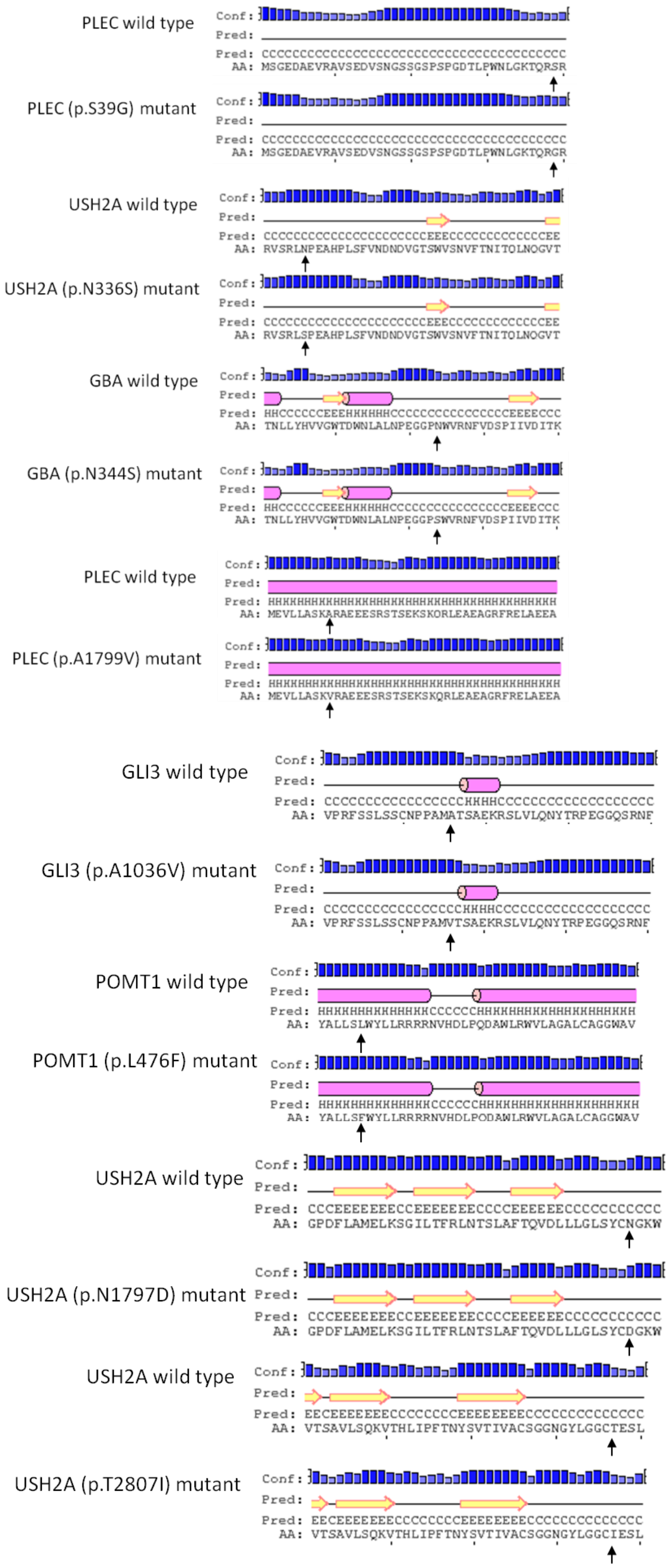

PLEC wild type
AA: MSGEDAEVRAVSEDVSNGSSGSPSPGDTLPWNLGKTQRSR
PLEC (p.S39G) mutant
AA: MSGEDAEVRAVSEDVSNGSSGSPSPGDTLPWNLGKTQRGR
USH2A wild type
AA: RVSRLNPEAHPLSFVNDNDVGTSWVSNVFTNITQLNQGVT
USH2A (p.N336S) mutant
AA: RVSRLSPEAHPLSFVNDNDVGTSWVSNVFTNITQLNQGVT
GBA wild type
AA: TNLLYHVVGWTDWNLALNPEGGPNWVRNFVDSPIIVDITK
GBA (p.N344S) mutant
AA: TNLLYHVVGWTDWNLALNPEGGPSWVRNFVDSPIIVDITK
PLEC wild type
AA: MEVLLASKARAEEESRSTSEKSKQRLEAEAGRFRELAEEA
PLEC (p.A1799V) mutant
AA: MEVLLASKVRAEEESRSTSEKSKQRLEAEAGRFRELAEEA
GLI3 wild type
AA: VPRFSSLSSCNPPAMATSAEKRSLVLQNYTRPEGGQSRNF
GLI3 (p.A1036V) mutant
AA: VPRFSSLSSCNPPAMVTSAEKRSLVLQNYTRPEGGQSRNF
POMT1 wild type
AA: YALLSLWYLLRRRRNVHDLPQDAWLRWVLAGALCAGGWAV
POMT1 (p.L476F) mutant
AA: YALLSFWYLLRRRRNVHDLPQDAWLRWVLAGALCAGGWAV
USH2A wild type
AA: GPDFLAMELKSGILTFRLNTSLAFTQVDLLLGLSYCNGKW
USH2A (p.N1797D) mutant
AA: GPDFLAMELKSGILTFRLNTSLAFTQVDLLLGLSYCDGKW
USH2A wild type
AA: VTSAVLSQKVTHLIPFTNYSVTIVACSGGNGYLGGCTESL
USH2A (p.T2807I) mutant
AA: VTSAVLSQKVTHLIPFTNYSVTIVACSGGNGYLGGCIESL

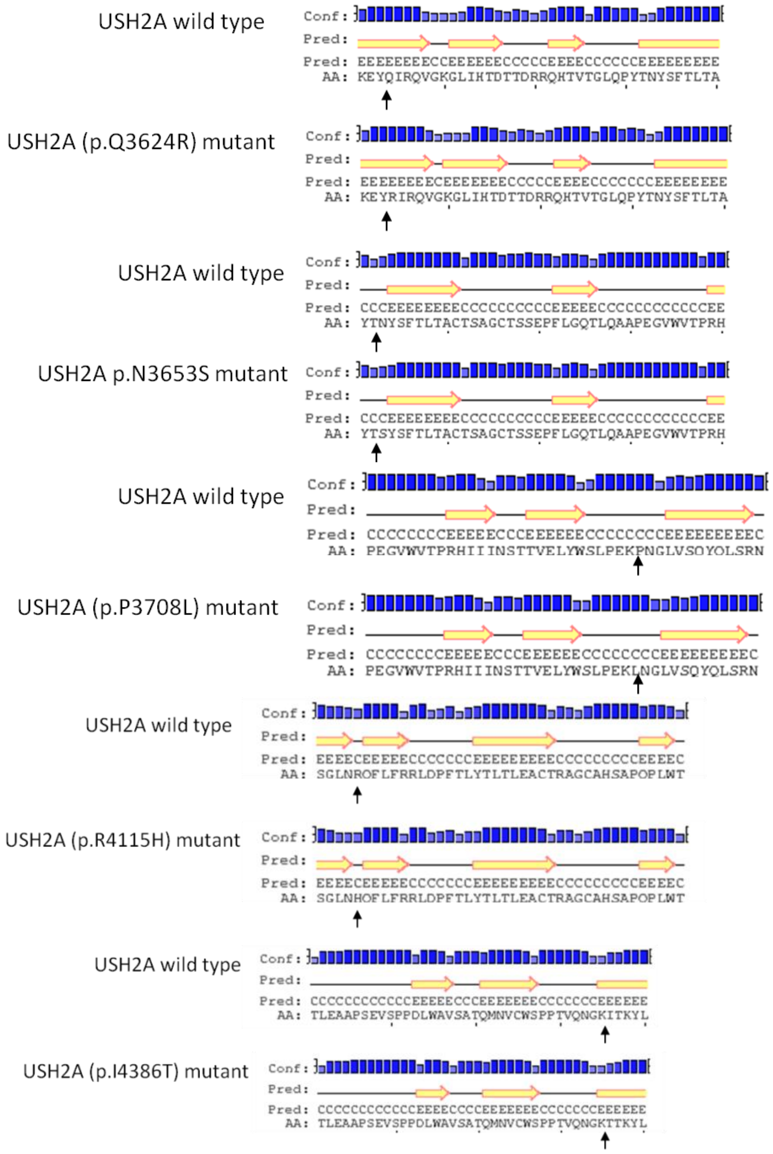
USH2A wild type
USH2A (p.Q3624R) mutant
USH2A wild type
USH2A p.N3653S mutant
USH2A wild type
USH2A (p.P3708L) mutant
USH2A wild type
USH2A (p.R4115H) mutant
USH2A wild type
USH2A (p.I4386T) mutant
